# Inhibition of NAD(P)H oxidase reduces apoptosis and avascular retina in an animal model of retinopathy of prematurity

**Published:** 2007-06-12

**Authors:** Yuta Saito, Pete Geisen, Abhineet Uppal, M Elizabeth Hartnett

**Affiliations:** Department of Ophthalmology, 6109A Neuroscience Research Building, University of North Carolina, Chapel Hill, NC

## Abstract

**Purpose:**

To study the mechanisms of action of the antioxidants, n-acetylcysteine (NAC), and the nicotinamide adenine dinucleotide phosphate (NAPDH) oxidase oxidase inhibitor, apocynin, on intravitreous neovascularization (IVNV), and retinal avascularity in a rat model of retinopathy of prematurity (ROP).

**Methods:**

Newborn rats exposed to oxygen-induced retinopathy underwent intraperitoneal (IP) injections of NAC (150 mg/kg) at post-natal day (p)2, p6, p10 (early NAC-treated), or p12 through p17 (late NAC-treated), apocynin (10 mg/kg) from p12 through p17, or phosphate buffered saline (PBS; controls). Lipid hydroperoxide (LHP) was measured in early NAC-treated oxygen-induced retinopathy (OIR) at p7, p14 and p18. Pups were placed in room air at p14. At p18, retinal flat mounts were scored for IVNV and avascular/total retinal area, or retinas were assayed for cleaved caspase-3 and vascular endothelial growth factor (VEGF) protein. In non-injected OIR pups, retinas were assayed for gp91^phox^. Cryosections were stained with isolectin B4, cleaved caspase-3, CD68, CD31, gp91^phox^, neuron-glial antigen 2 (NG-2), or anti-glial fibrillary acidic protein (GFAP) and visualized with confocal microscopy.

**Results:**

LHP increased over time in retinas from OIR exposed pups in association with IVNV. Early NAC-treated retinas had significantly reduced LHP compared to PBS-control at p18 (p<0.012). However, neither early nor late treatment with NAC had an effect on IVNV or retinal avascularity. Although apocynin had no effect on IVNV, it reduced both avascular retina (p=0.017) and retinal cleaved caspase-3 determined by western blot (p=0.021). In cryosections from OIR eyes, cleaved caspase-3 positive cells co-labeled with some lectin-stained vessels, NG2 labeled cells, and with GFAP positive cells in the inner nuclear layer. We found that the intravascular expression of gp91^phox^ co-localized mostly with CD31 and some CD68 positive cells.

**Conclusions:**

Our results do not support the antioxidant properties of NAC as effective in reducing IVNV or avascular retina in the 50/10 OIR rat model. Apocynin reduced avascularity and apoptosis in the OIR model perhaps through pathways triggered by ROS generation but upstream from LHP production. Further study and consideration may be given to apocynin or NAD(P)H oxidase inhibitors as adjunctive therapy for ROP to reduce the avascular retina.

## Introduction

Oxidative stress has been linked to retinopathy of prematurity (ROP) through several mechanisms related to oxygenation of retinal tissue [1-4]. The retina is believed to be vulnerable to oxidative damage because of the abundance of polyunsaturated fatty acids, the high metabolic rate, and rapid rate of oxygen consumption of the photoreceptors, and possibly from light-induced free radical formation [5-7]. In addition, the premature infant has reduced ability to scavenge reactive oxidative species [8].

Both clinical and basic research support oxidative compounds as playing a role in ROP. A meta-analysis of several clinical studies that tested vitamin E to reduce ROP in infants found a 52% overall reduction in the incidence of stage 3 ROP, i.e. the stage with intravitreous neovascularization (IVNV). The study was not able to evaluate later visual development or complications of ROP [9]. In an oxygen-induced retinopathy (OIR) model in rat, treatment with forms of vitamin E [10,11] or manganese-superoxide dismutase [12] did not reduce pathologic IVNV but, compared to sham-injected controls, did reduce the area of avascular retina, which, in human disease has been associated with greater risk of poor visual outcomes [13]. The avascular retina is proposed as the stimulus for IVNV through the release of angiogenic growth factors, such as vascular endothelial growth factor (VEGF). Reducing the size of avascular retina may help to reduce vision loss in ROP, both by reducing the stimulus for IVNV and by increasing the area of vascularized retina. However, despite the lines of evidence supporting the role of oxidative stress in ROP, to date, treatment of ROP with antioxidants has not been widely accepted or successful [14]. This in part is because of the complexities in studying oxidative pathways, the difficulty in studying ROP in human infants, and the need for effective yet safe treatments in the developing infant.

Although there are several models of OIR that have been studied [4,15-22], the Penn 50/10 OIR model is most relevant to what the human preterm infant with ROP currently experiences [23], namely: (1) fluctuations in oxygen rather than constant oxygen [4,24]; (2) a similarity in the extremes of oxygen fluctuations that a preterm infant who developed severe ROP experienced [23]; (3) retinal features similar to those of human ROP [25]. We proposed that the changes in tissue oxygenation associated with fluctuations in inspired oxygen [26] might lead to an increase in oxidative substances particularly at the vulnerable junction of vascular and avascular retina where the contrast in tissue oxygen levels is likely greatest. Oxidative stress could then lead to several pathologic events in ROP, including apoptosis of nascent intraretinal vascularization or pathologic IVNV [27] by triggering signaling of VEGF, inflammatory pathways, and TNF-α or other cytokines [28]. To understand the role of oxidative compounds in ROP development we used the 50/10 OIR model and analyzed for lipid hydroperoxides (LHP), which are end-products of oxidative stress and themselves causative of IVNV [29], and for gp91^phox^, a subunit of NAD(P)H oxidase, which can increase the release of reactive oxygen species and trigger many signaling pathways including apoptotic and inflammatory pathways [30]. NAD(P)H oxidase can also increase VEGF expression, trigger signaling pathways causing endothelial migration [31], and has been associated with neovascularization in some models [31,32]. Greater knowledge of the mechanisms of oxidative compounds in a relevant model system may enable the development of directed and safe therapies for ROP.

## Methods

All animals were cared for in accordance with the University of North Carolina's Institute for Laboratory Animal Research (Guide for the Care and Use of Laboratory Animals) and the ARVO Statement for the Use of Animals in Ophthalmic and Vision Research.

### Model of oxygen induced retinopathy

A bioactive gas controller (Oxycycler; BioSpherix, New York, NY), which regulates the atmosphere inside an incubator by injecting either nitrogen or oxygen, was used to induce OIR in newborn Sprague-Dawley rats (Charles River, Wilmington, MA) as reported [33]. Within 24 h of birth, pups designated postnatal day (p)0 and their mothers were placed into the incubator. Oxygen was cycled between 50% and 10% every 24 h for 14 days, and then the pups were returned to room air for four additional days (50/10 OIR model). Litters of 12 to 14 pups were used in all experiments.

### Inhibition of oxidative compounds

We studied the effects of n-acetylcysteine (NAC, 150 mg/kg, Sigma, St. Louis, MO), a water soluble antioxidant [34,35] in the OIR model. NAC was diluted in phosphate buffered saline (PBS: 7 g NaCl, 3.4 g Na_2_HPO_4_, 0.8 g KH_2_PO_4_, 1 l diH_2_O) injected IP at different time points in two experimental groups. In the early group, pups received IP injections at p2, p6, and p10 and in the later groups daily from p12 to p17. The choice of drug regimen for each group was based on reducing avascular retina (early NAC treated group) or IVNV (late NAC treated group) without greatly deviating from the oxygen cycling regimen, which can affect model outcomes [25]. Controls received IP injections of PBS. To inhibit NAD(P)H oxidase, apocynin (Sigma) diluted in PBS was injected IP (10 mg/kg) daily from p12 through p17. Controls received IP injections of PBS at the same time points.

### Dissecting retinal tissue for flat mounting and cryosections

Pups were deeply anesthetized by IP injection of ketamine (60 mg/kg) and xylazine (18 mg/kg). One ml of 0.5% paraformaldehyde (PFA, Sigma) in PBS was then directly perfused by intracardiac injection into the left ventricle before euthanizing by intracardiac injection of pentobarbital (80 mg/kg). Both eyes were enucleated and fixed in 2% PFA for 2 h. The anterior segments were removed, and the retinas with the ora serratas intact were carefully dissected and placed into PBS, with care to remove the hyaloidal vessels and any remaining vitreous. For flat mounts, each retina was then placed onto a microscope slide and flattened by making four incisions, each 90 ° apart, beginning at the ora serrata and extending centrally from the equator, stopping short of the optic nerve opening. For cryosections, eyes with anterior segments removed were put into 30% sucrose in PBS overnight. Each eye was dried with filter paper, soaked in optimal cutting temperature (O.C.T., Tissue-Tek, Torrance, CA) and kept at -80 °C for future sectioning, immunostaining and analysis.

### Tissue staining for flat mounts

The flattened retinas were permeabilized in ice-cold 70% (vol/vol) ethanol for 20 min and then in PBS/1% Triton X-100 for 30 min. The retinas were incubated with Alexa Fluor 568-conjugated *Griffonia simplicifolia* (Bandeiraea) isolectin B4 (5 μg/ml; Invitrogen, Carlsbad, CA) in PBS overnight at 4 °C for staining of the vasculature. The retinas were rinsed three times in PBS and mounted in PBS-glycerol (2:1, VectaShield; Vector Laboratories), and the coverslips sealed with nail polish. Images of the superficial blood vessel layers were captured with an inverted microscope (TE2000U, Nikon, Tokyo Japan; Michael Hooker Microscopy Facility, University of North Carolina, Chapel Hill) and digitally stored for analysis. Digitized image sections were stitched together into a montage of the retinal flat mount using a commercial image-management software (Adobe Photoshop 7.0: Adobe Systems, Mountain View, CA).

### Counting clock hours of neovascularization

Retinal images were randomized and divided into 12 clock hours of approximately equal area by using image-analysis software (Photoshop 7.0; Adobe Systems, Mountain View, CA). Each clock hour was then assessed by a masked examiner for the presence of IVNV using an assessment technique adapted from those used in animal models to quantify vessel tufts [36]. Each retina received a score of 0 to 12, based on the number of clock hours exhibiting IVNV.

### Analysis of peripheral avascular areas

Digitized images of the total retinal area and peripheral avascular areas were measured using Image J software (NIH, Bethesda, MD). The peripheral avascular area was expressed as a percentage of the total retinal area.

### Quantification of capillary density

Central capillary density was quantified by summing capillary junctions within 4 equal square areas, each 0.16 mm^2^, in each of the 4 quadrants of the vascularized retina and expressed as number of junctions per 0.64 mm^2^ using ImageTool, Version 3 (University of Texas, San Antonio, TX).

### Tissue staining for cryosections

Retinas frozen in O.C.T. were cut into 10 μm sections and incubated in PBS/1% Triton X-100 for 30 min. Some sections were incubated with Alexa Fluor 568-conjugated *Griffonia simplicifolia* (Bandeiraea) isolectin B4 (Invitrogen, Carlsbad, CA) in PBS for 30 min at room temperature to stain the vasculature. After washing in PBS three times, sections were incubated for 30 min in 3% normal goat serum to block non-specific binding. Anti-gp91^phox^ rabbit antibody (Santa Cruz Biotechnology, Santa Cruz, CA), anti-rat CD68 mouse antibody (Serotec, Raleigh, NC), NG2 mouse antibody (Upstate, Charlottesville, VA), anti-rat CD31 mouse antibody (Serotec, Raleigh, NC) and cleaved caspase-3 rabbit antibody (Cell Signaling Technology, Danvers, MA) were used at a dilution of 1:100 in PBS and the sections incubated for 60 min at room temperature. After three washes in PBS, the sections were incubated for 20 min with a 1:500 dilution of a goat anti-mouse Alexa 568 secondary antibody (Invitrogen) for CD68, NG2 and CD31 or goat anti-rabbit Alexa 488 secondary antibody (Invitrogen) for cleaved caspase-3 and gp91^phox^. Some sections were incubated with Alexa Fluor 594-conjugated anti-glial fibrillary acidic protein (GFAP) mouse antibody (Invitrogen) in PBS for 60 min at room temperature. The sections were rinsed three times in PBS, then incubated in a 1:5,000 dilution of Hoechst 33342 (Invitrogen) for 15 min and mounted in PBS-glycerol (2:1 with VectaShield; Vector Laboratories, Burlingame, CA). Negative controls used were samples incubated in conjugated secondary antibody only. The cover slips were sealed with nail polish. Images of the sections were captured with a scanning laser confocal microscope (Leica SP2, Wetzlar, Germany) and digitally stored for analysis.

### Fresh tissue preparation

Animals were euthanized with an overdose of pentobarbital (80 mg/kg) injected IP. Eyes were enucleated and the retinas were isolated under a dissecting microscope in a similar fashion as used for flat mounting except that the ora serratas were carefully removed. The tissue was placed in HPLC-grade water for the LHP assay or RIPA buffer (20 mM Tris base, 120 mM NaCl, 1% Triton X-100, 0.5% sodium deoxycholate, 0.1% sodium dodecyl sulfate (SDS), 10% glycerol) with a protease inhibitor cocktail (1:100, Sigma) for western blot and ELISA.

### Determination of retinal lipid hydroperoxide

Retinas from pups in the OIR model injected IP with NAC at early time points (p2, 6, 10) were measured for LHP at p7, p14, and p18 and compared to PBS injected OIR pups at similar time points. The LHP assay was performed according to the manufacturer's protocol (Lipid Hydroperoxide Assay kit, Cayman Chemical, Ann Arbor, MI). Briefly, freshly dissected unfixed retinal tissues in HPLC-grade water were homogenized and the supernatant fractions from homogenized retinas centrifuged at 13,000xg for 15 min at 4 °C were extracted with 0.25 ml of Extract R (Lipid Hydroperoxide Assay kit) saturated methanol (methanol was degassed with nitrogen for at least 30 min) and mixed by vortexing for 30 s. One milliliter of degassed chloroform was added to each polypropylene tube and mixed by vortexing for 30 s. The samples were centrifuged at 13,000xg for 5 min at 4 °C. The lower chloroform phase was transferred to clean polypropylene tubes and stored at -80 °C for future analysis. The standard LHP preparation and chromogenic reaction assay was done according to the manufacturer's protocol. After color development, the samples were pipetted into the wells of a 96-well glass plate and absorbance with measured with a FLUOstar OPTIMA (BMG LABTECH, Offenburg, Germany) at 492 nm. The Bradford assay (Bio-Rad, Hercules, CA) was performed to determine the protein concentration of the cell lysate. LHP was expressed in nmol LHP/mg total protein.

### Protein extraction and western blot analysis

Freshly dissected unfixed retinal tissue immersed in RIPA buffer was homogenized, and lysates were centrifuged at 13,000xg for 15 min at 4 °C. The supernatants were collected, and the Bradford assay was performed to determine protein concentration of the cell lysate. The cell lysates were eluted with 6x sample buffer (2.4 ml 1M Tris-HCl pH 6.8, 0.96g SDS, 4.8 ml glycerol, 759 mg DTT, 4.8 mg bromophenol blue). 50 μg protein samples were separated by 7.5% or 15% sodium dodecyl sulfate-polyacrylamide gel electrophoresis, transferred to a polyvinylidene difluoride (PVDF) membrane, and reacted with gp91^phox^ antibody (1:1000, BD Transduction Laboratories, San Diego, CA), cleaved caspase-3 antibody (1:1000, Cell Signaling Technology, Danvers, MA) or VEGF antibody (1:1000, Santa Cruz Biotechnology, Santa Cruz, CA) overnight, followed by goat ant-rabbit HRP conjugated secondary (1:1000, R&D Systems, Minneapolis, MN) for cleaved caspase-3 and VEGF or goat anti-mouse HRP conjugated secondary antibody (CHEMICON, Temecula, CA) for gp91^phox^. β-actin (1:10000, Abcam Cambridge, MA) was measured as a control. Visualization was performed with enhanced chemiluminescence (Pierce, Rockford, IL). The signal intensity was quantified from exposed films with analysis software (UN-SCAN-IT, ver.6.1; Silk Scientific, Orem, UT).

### Statistical analysis

Data were analyzed with software (SPSS version 14.0 Chicago, IL). Analysis of variance and t tests were used to analyze parametric data such as avascular area/total retinal area, capillary density, and normalized densitometric values. Non-parametric data for IVNV score obtained by the clock-hour method were analyzed with the Mann-Whitney test. For each test, p<0.05 was considered significant. All western blots had at least an N of 4.

## Results

### Lipid hydroperoxides increased in 50/10 oxygen-induced retinopathy model in association with intravitreous neovascularization and avascular retina

**Figure 1:**
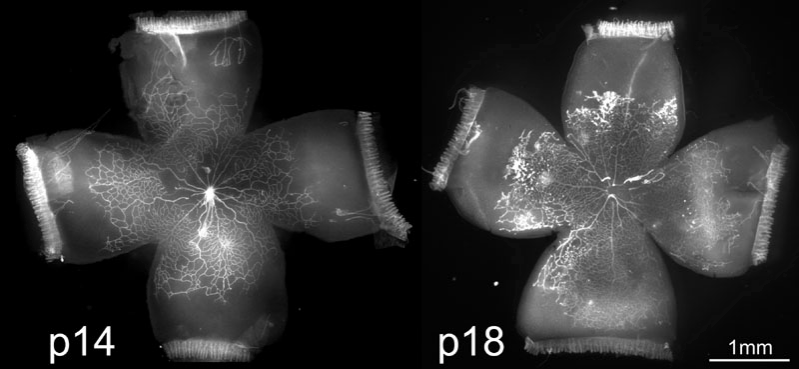
Rat 50/10 oxygen-induced retinopathy model. Rat retinal flat mounts stained with lectin at p14 (**A**) and p18 (**B**). Note peripheral avascular areas of retina at p14 after 7 cycles of oxygen fluctuations (**A**) and IVNV at the junction of vascular and avascular retina at p18 after 4 days of RA exposure (**B**).

**Figure 2:**
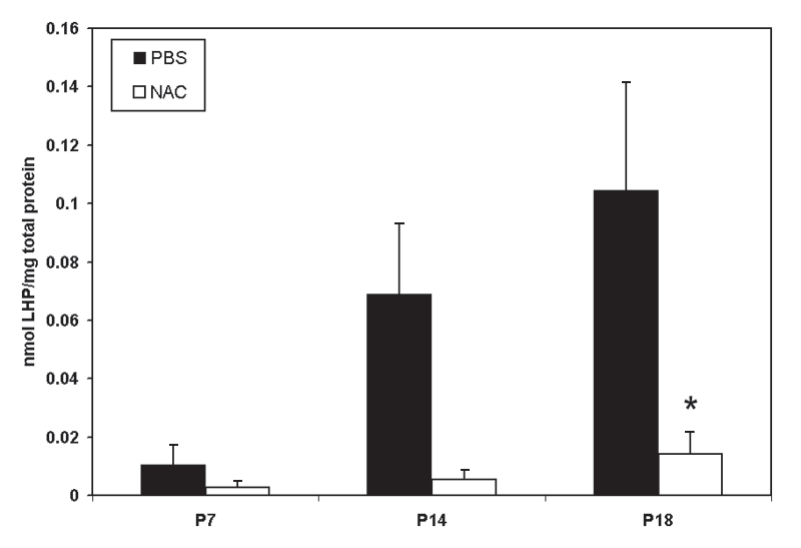
Lipid hydroperoxide in 50/10 oxygen-induced retinopathy model. Lipid hydroperoxide (LHP) levels in retinas assayed at postnatal days 7, 14, or 18 (p7, p14, p18) from pups injected intraperitoneally (IP) with n-acetylcysteine (NAC) or PBS at postnatal days p2, p6, and p10 in the 50/10 oxygen-induced retinopathy (OIR) model. Overall ANOVA, p<0.001, * p=0.012, Bonferroni post-hoc analysis comparing p18 NAC to p18 PBS injected. (n=8 animals at p7 and 4 animals at p14 and p18).

The 50/10 OIR model developed peripheral avascular retina at p14, after 7 cycles of oxygen fluctuations, followed by IVNV at the junctions of vascular and avascular retina at p18, as previously published [37] (Figure 1). Since oxidative stress has been linked to ROP, we wished to determine whether there was increased production of oxidative compounds in the 50/10 OIR model in association with IVNV occurrence. We measured lipid hydroperoxide (LHP), end-products of oxidation, in whole retinas from rat pups in the OIR model. LHP increased in the model over time (Figure 2, PBS-injected pups) at the time of maximal IVNV.

### Inhibition of lipid hydroperoxides does not reduce avascular retina or intravitreous neovascularization in 50/10 oxygen-induced retinopathy model

**Figure 3:**
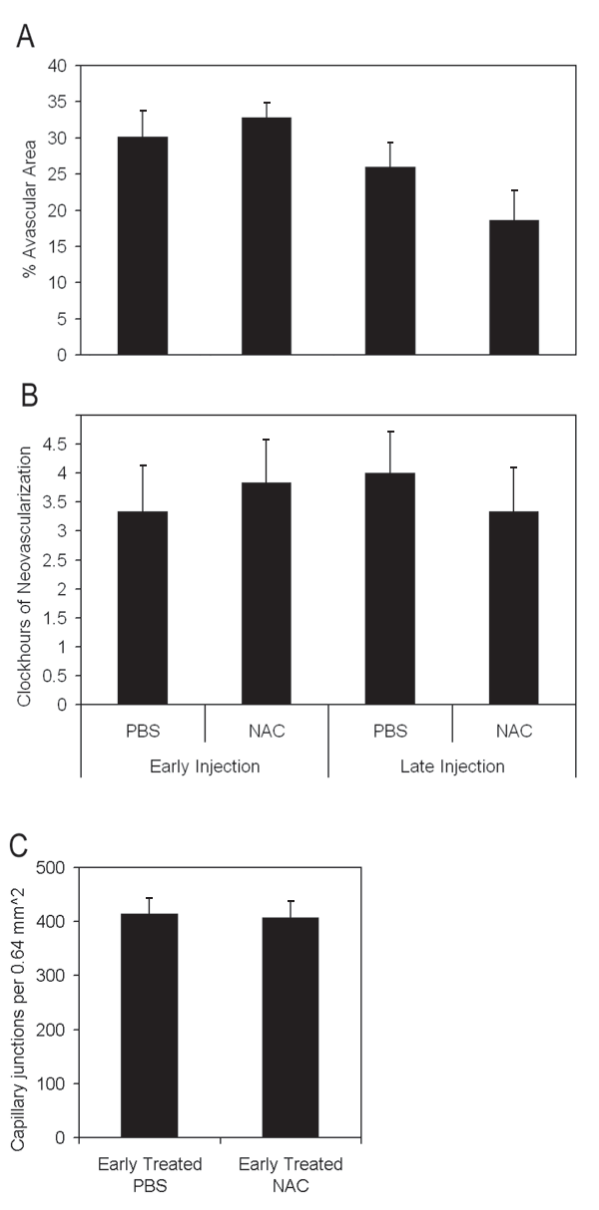
Avascular retinal area and clock hours of intravitreous neovascularization and capillary densities of n-acetylcysteine treated pups. **A**: Avascular retinal areas from pups exposed to 50/10 oxygen-induced retinopathy (OIR) that had intraperitoneal (IP) injections of n-acetylcysteine (NAC) (n=6 each) at p2, p6, and p10 (early treatment group) or once every 24 h from p12-p17 (n=6 each; late treatment group) and assayed at p18 showed no significant difference compared to respective PBS controls (n=6 each; early, p=0.54; late, p=0.19; Student t-test). **B**: Clock hours of intravitreous neovascularization (IVNV) in retinas of OIR pups after IP injections of NAC (n=6 each) at p2, p6, and p10 (early treatment group) or once every 24 h from p12-p17 (n=6 each; late treatment group) and assayed at p18 showed no significant difference compared to respective PBS controls (n=6 each; early, p=0.56; late, p=0.51; Mann-Whitney test). **C**: Capillary densities did not differ between NAC- or PBS- injected early treatment groups (p=0.88; n=6, Student t-test).

**Figure 4:**
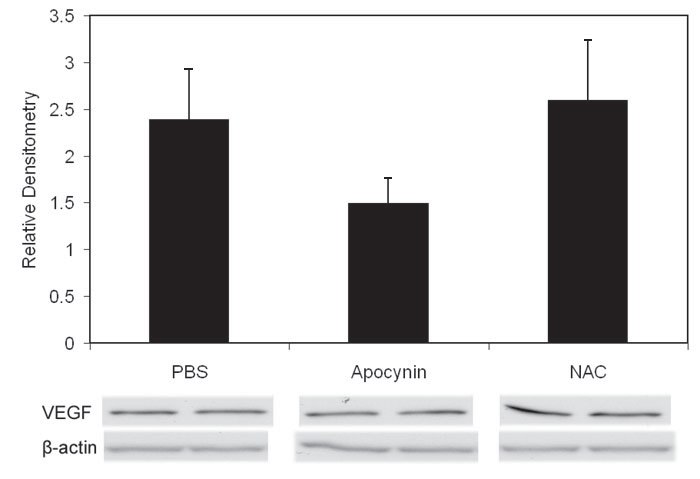
Western blots of vascular endothelial growth factor from p18 retinas of rats Injected with n-acetylcysteine, apocynin, or phosphate buffered saline. Western blot of vascular endothelial growth factor (VEGF) in oxygen-induced retinopathy pups treated with PBS, apocynin, and n-acetylcysteine (NAC; intraperitoneal injections once every 24 h from p12 to p17) showed no difference in VEGF protein from retinas analyzed at p18 (p=0.82, Student t-test, n=4, phosphate buffered saline (PBS) versus NAC; p=0.201, Student t-test, n=4, PBS versus apocynin). Overall ANOVA comparing three groups p=0.3120.

**Figure 5:**
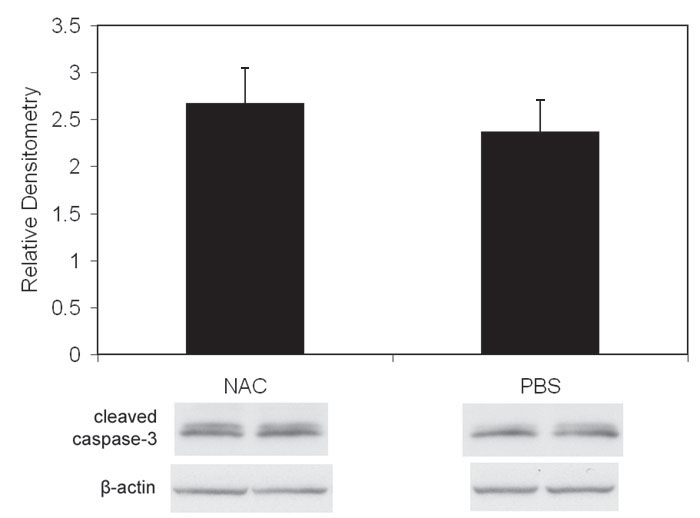
Western blot of cleaved caspase-3 from p18 retinas of pups injected with n-acetylcysteine or phosphate buffered saline. Western blot of cleaved caspase-3 showed no difference in retinas from OIR pups that received intraperitoneal injections once every 24 h from p12 to p17 of n-acetylcysteine versus phosphate buffered saline (p=0.74, Student t-test, n=4).

Since oxidative compounds can trigger pathways of angiogenesis [30] or apoptosis [38] directly or through release of inflammatory cytokines [39], we inhibited LHP production using NAC and determined the effect on avascular retina or IVNV. NAC was injected at two different time points in separate regimens: early (with the rationale of reducing apoptosis and avascular retina) and later at the time of greater LHP production (with the rationale of reducing IVNV). NAC significantly reduced LHP in the OIR model (Figure 2: ANOVA p<0.001, p7 (n=8); p14, and p18 [n=4 each]). However, despite significantly inhibiting the production of LHP with the dose of NAC used, there was no significant difference in peripheral avascular area (Figure 3A, p=0.54 (early treatment), p=0.19 (late treatment), Student t-Test, n=6) or IVNV (Figure 3B, p=0.56 (early treatment), p= 0.51 (late treatment), Mann-Whitney test, n=6) from early NAC-treated groups or late NAC-treated groups compared to respective PBS controls. In addition, there was no difference in capillary densities between NAC- and PBS-injected retinas from the early treatment group (Figure 3C, p=0.88, n=6, Student t-test). At p18, there was no difference in the amount of VEGF protein (Figure 4, p=0.82, n=4, Student t-test), or cleaved caspase-3 (Figure 5, p=0.74, n=4, Student t-test) measured in retinas from late NAC treated or PBS treated pups by western blot.

### gp91^phox^ expression in 50/10 oxygen-induced retinopathy model

**Figure 6:**
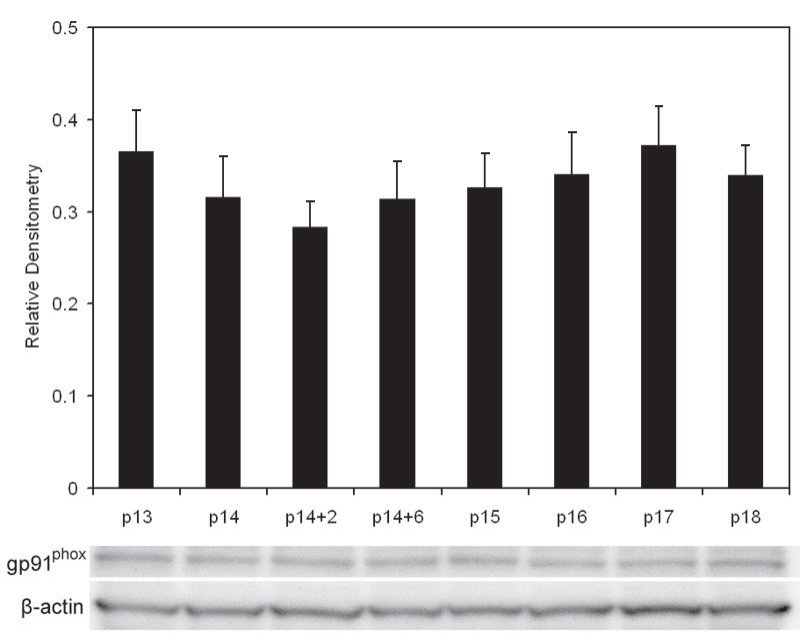
p91^phox^ expression by western blot in oxygen-induced retinopathy retinas. P14 pups were euthanized immediately after removal from hypoxia (p14), after 2 h of room air (p14+2), after 6 h of room air (p14+6) or at p15, p16, p17, p18. Values are expressed as gp91^phox^ over β-actin (n=at least 4 samples per group). gp91^phox^ expression did not significantly change over the time points measured (p=0.99, ANOVA, n=at least 4/time point).

We also studied a gp91^phox^ (NOX2) containing NAD(P)H oxidase. NOX family members are transmembrane proteins that transport electrons across biological membranes to reduce oxygen and are selective for NADPH over NADH. gp91^phox^ is one of the most studied of the NOX family members. Phagocytes and endothelial cells can express gp91^phox^ and endothelial cell NAD(P)H oxidase can trigger pathways involving endothelial cell migration and VEGF expression, as well as inflammatory, apoptotic, and oxidative pathways [30,31,39]. Because these pathways may be important in reducing retinal avascularity or IVNV independent of LHP formation, we measured gp91^phox^ at relevant time points around the transition from avascular retina (p14) to IVNV (p18) in the 50/10 OIR model. The variation in gp91^phox^ protein/actin in whole retinas was not significant at time points measured (Figure 6, p=0.99, ANOVA, n=at least 4 per time point).

### Apocynin reduces retinal avascularity but not intravitreous neovascularization

**Figure 7:**
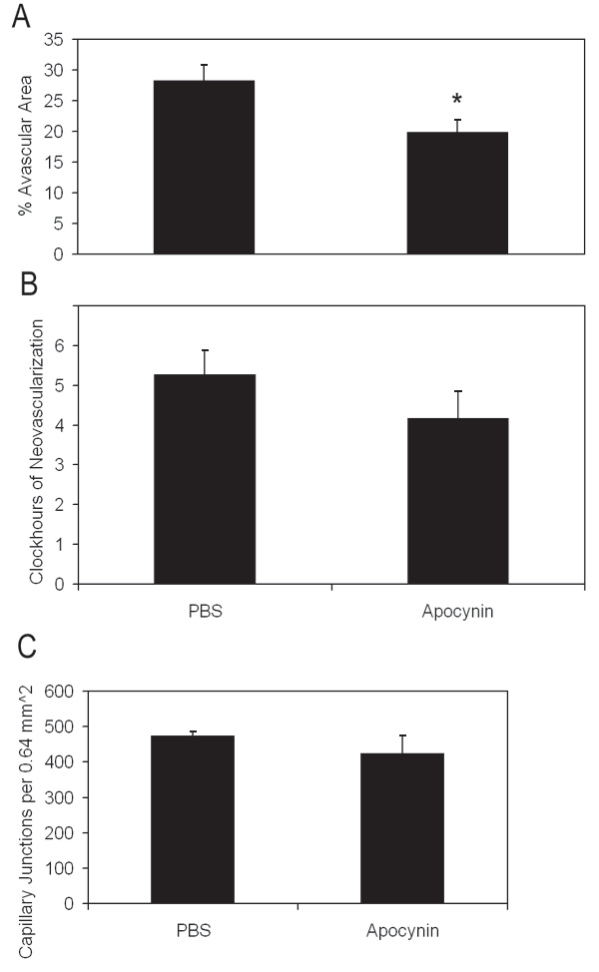
Avascular retinal area and clock hours of intravitreous neovascularization and capillary densities of apocynin treated pups. **A**: Percent avascular retinal area in oxygen-induced retinopathy (OIR) after intraperitoneal (IP) injections with apocynin once every 24 h from p12 to p17 and assayed at p18 was significantly reduced compared to phosphate buffered saline (PBS) injected pups (* p=0.017, Student's t-test; n=11 retinas each). **B**: Clock hours of intravitreous neovascularization (IVNV) in OIR retinas after IP injections with apocynin once every 24 h from p12 to p17 and assayed at p18 were not significantly different to PBS injected pups (p=0.26; Mann-Whitney test, n=11). **C**: At p18, capillary densities did not differ between apocynin and PBS injected treatment groups. (p=0.37; Student t-test, n=6).

Although inhibition of LHP with NAC did not reduce retinal avascularity or IVNV, LHP increased in the 50/10 OIR model in association with these features. NAD(P)H oxidase can cause a release of reactive oxygen species (ROS), which ultimately can lead to the end products, LHP. Through ROS generated prior to LHP production, NAD(P)H oxidase can also cause apoptosis, inflammation, and changes in cell proliferation. In addition, it can cause apoptosis more directly by activation of proapoptotic signaling proteins [30]. Therefore, we determined whether inhibition of NAD(P)H oxidase would reduce IVNV or avascular retina perhaps through a mechanism parallel to or upstream from LHP production. Based on our data of increased oxidative compounds at p14 and p18 (Figure 1), we injected the NAD(P)H oxidase inhibitor, apocynin, IP daily from p12 to p17, similar to the NAC late-treatment group. We found that apocynin-injected groups had significantly smaller peripheral avascular areas compared to controls (Figure 7A, Student's t-test p=0.017, n=11). However, there was no significant difference in IVNV score (Figure 7B, p=0.26, Mann Whitney test, n=11).

### Apocynin inhibits apoptosis but not vascular endothelial growth factor expression

**Figure 8:**
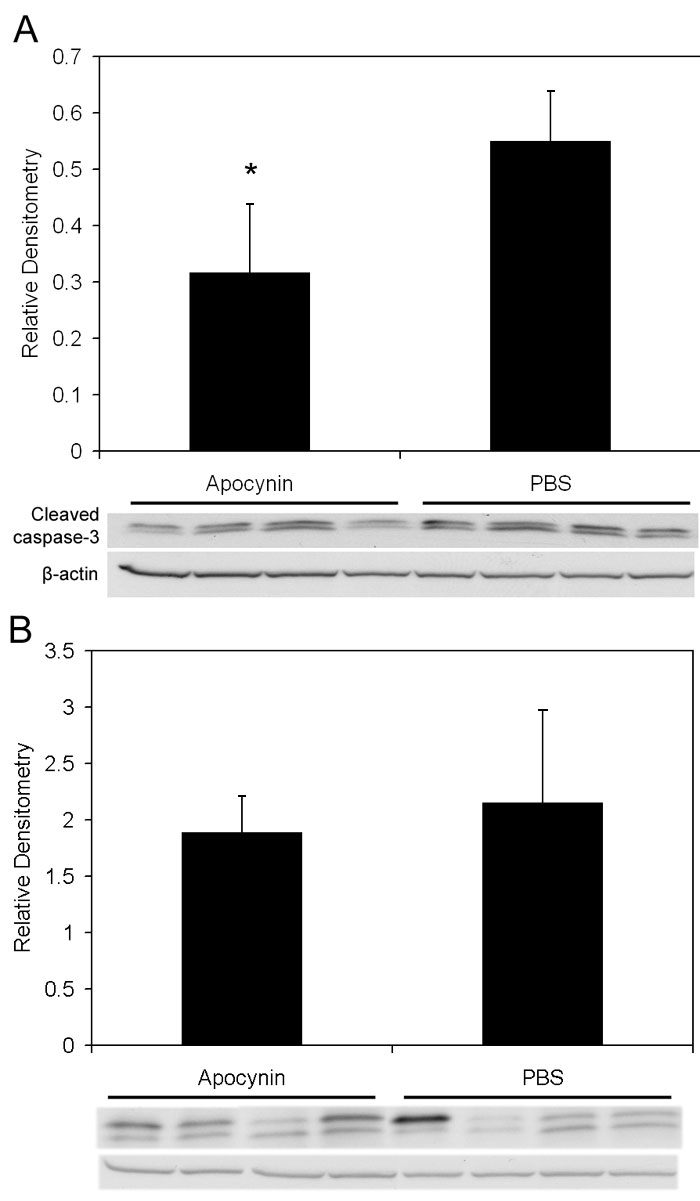
Cleaved caspase-3 in retinas from apocynin treated pups. **A**: Cleaved caspase-3 expression in the retinas of OIR after IP injection with apocynin on p12 and p13 and assayed on p14 was significantly decreased compared to retinas from PBS injected pups (p=0.021, Student's t-test, n=4). **B**: However, no significant difference in cleaved caspase-3 expression was measured in retinas of pups injected with apocynin every 24 h from p12 to 17 and assayed on p18 compared to PBS injected pups (p=0.78, Student t-test, n=4).

Since the avascular retina was reduced, we wished to determine whether angiogenic or apoptotic pathways were affected by apocynin. Therefore, we measured VEGF expression, which is a survival factor for endothelial cells and neurons, and cleaved caspase-3, a cysteine protease expressed during apoptosis, in whole retinas from PBS and apocynin treated pups. There was no significant difference in VEGF protein measured at p18 in apocynin injected groups compared to PBS injected determined by western blot (Figure 4; p=0.77, n=4). There was also no difference in capillary density of newly vascularized retinas between apocynin and PBS treated groups (Figure 7C, p=0.37, Student t-Test, n=6). However, apocynin significantly inhibited cleaved caspase-3 determined by western blot of whole retinas as compared to controls at p14 (Figure 8A, Student's t-test p=0.021, n=4) but not at p18 (Figure 8B, Student t-test, p=0.78, n=4).

### 50/10 OIR Model shows apoptosis of endothelial cells, pericytes, and glial cells and expression of endothelial cell gp91^phox^

**Figure 9:**
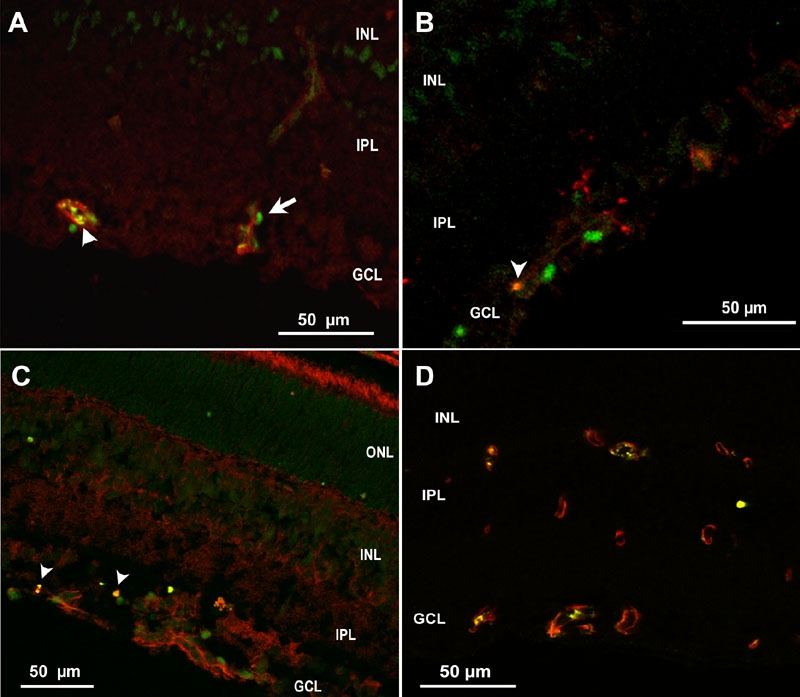
Cryosection of p18 oxygen-induced retinopathy eye from pups after intraperitoneal injection with phosphate buffered saline from p12 to p17. **A**: Cleaved caspase-3+ cells (green) were adjacent to lectin stained vessels (red), suggesting possible pericyte or leukocyte apoptosis, whereas lectin also co-stained with cleaved caspase-3 (arrowhead), supporting apoptosis of endothelial cells. **B**: Rare CD68+ stained cells (red) co-labeled with cleaved caspase-3 (Green) (arrowhead). **C**: Cleaved caspase-3+ nuclei in inner nuclear layer (INL) and ganglion cell layer (GCL, green), some of which only rarely co-labeled with anti-glial fibrillary acidic protein (GFAP, red arrowhead), suggesting apoptosis of glia. **D**: Mostly CD31+ endothelial cells (red) co-localized with gp91^phox^ (green) in vessels. ONL represents outer nuclear layer; IPL represents inner plexiform layer.

**Figure 10:**
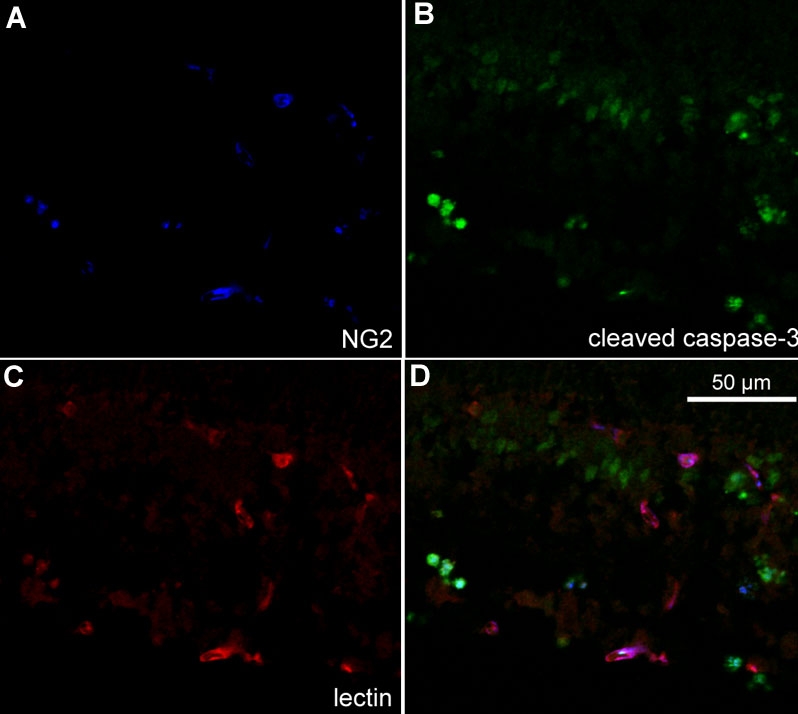
Cryosection of p18 oxygen-induced retinopathy eye from pups after intraperitoneal injection with phosphate buffered saline from p12 to p17. Labeling with NG2 (**A**, blue), cleaved caspase-3 (**B**, green), and lectin (**C**, red). Some NG2 labeled cells co-localized with cleaved caspase-3 (aqua, **D**) suggesting pericyte apoptosis. GCL represents ganglion cell layer; INL represents inner nuclear layer; IPL represents inner plexiform layer.

**Figure 11:**
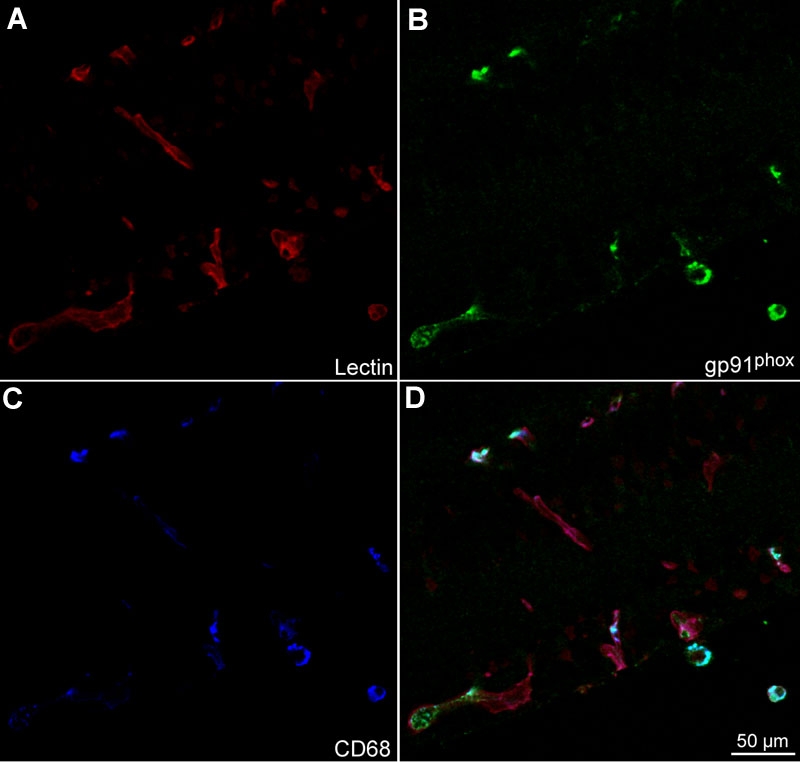
Cryosection of p18 oxygen-induced retinopathy eye from pups after intraperitoneal injections with phosphate buffered saline from p12 to p17. Labeling with lectin (**A**, red), gp91^phox^ (**B**, green), and CD68 (**C**, blue). gp91^phox^ co-labeled CD68 cells (**D**, light blue cells). CD68 stained leukocytes aligned with lectin stained vessels (**D**, magenta).

To characterize the cells that were apoptotic in the OIR model, we examined cryosections of p18 eyes from 50/10 OIR control eyes that were co-labeled with cleaved caspase-3 and lectin; CD68, lectin and cleaved caspase-3; lectin, NG-2, and cleaved caspase-3; or glial fibrillary acidic protein (GFAP) and cleaved caspase-3 using confocal microscopy. Cleaved caspase-3 positive cells were adjacent to lectin stained vessels (Figure 9A, arrow) and co-stained with NG-2 suggesting pericyte involvement (Figure 10). In some cases, lectin co-stained with cleaved caspase-3 (Figure 9A, arrowhead) providing evidence that some endothelial cells were apoptotic. CD68 labeling was greatest in the regions of retinal vasculature and less in the regions of avascular retina. CD68 stained cells and processes aligned with blood vessels in the inner nuclear layer adjacent to the outer plexiform layer and at the interface with the vitreous (Figure 11). Only rarely were CD68 positive cells co-labeled with cleaved caspase-3 (Figure 9B, arrowhead). Few GFAP positive nuclei co-stained with cleaved caspase-3 (Figure 9C, arrowhead). These results suggest that apoptosis occurred mainly in endothelial cells, perivascular cells representing pericytes, and possibly glia in the 50/10 OIR model at p18. These data, though sensitive, are qualitative and are viewed with the knowledge that apoptotic cells may lose cell markers making it difficult to identify them with immunohistochemical stains.

**Figure 12:**
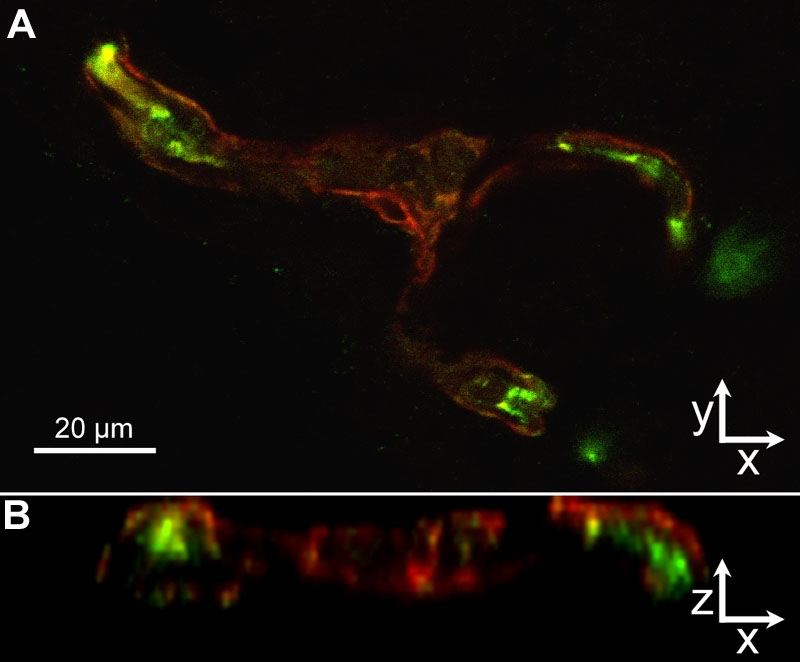
Cryosection of p18 oxygen-induced retinopathy eye from pups after intraperitoneal injections with phosphate buffered saline from p12 to p17. gp91^phox^ localization (**A**, green) resided mainly inside of the /lectin stained (red) /vessel wall (z-series, **B**), suggesting that endothelial cells, and not NG-2 positive pericytes (red), express NAD(P)H oxidase in the oxygen-induced retinopathy model.

To determine what cells stained for gp91^phox^, we qualitatively analyzed cryosections of retinas from control OIR rat retinas at p18. gp91^phox^ cells co-labeled with CD68 (Figure 11) suggesting a leukocyte origin. However, most of the staining at the time point analyzed was localized within the vessels. We co-labeled sections with CD31 or NG2 and gp91^phox^ and found that gp91^phox^ co-localized mostly with CD31+ cells (Figure 9D) and not with NG2+ pericytes (Figure 12), suggesting that endothelial cells account for a large proportion of the intravascular gp91^phox^ expression. There did not appear to be a difference in gp91^phox^ staining within intraretinal blood vessels located at the junction of vascular and avascular retina at p18 in OIR compared with blood vessels more centrally located.

## Discussion

Evidence supports oxidative compounds play a role in ROP [1-4]. The retina is susceptible to oxidative damage [6] given its high metabolic rate and rapid rate of oxygen consumption [5]. In addition, the premature infant has a reduced ability to scavenge reactive oxidative species [8], increasing its vulnerability to oxidative stress. We found that LHP were significantly increased in the 50/10 OIR model at time points corresponding to IVNV. LHP induce a cascade of interacting angiogenic cytokines [28] such as VEGF, TNF-α, IL-1α, PDGF, and TGF-β. When LHP are injected into the rabbit vitreous, they can cause IVNV [28]. Yet, our data suggest that other pathways may play a role in the pathogenesis of ROP and that endogenous LHP themselves are not causative of IVNV in the rat OIR model.

NAC has reducing and antioxidant properties by acting as a direct scavenger of free radicals including OH^-^, H_2_O_2_ and O2^-^ [40,41]. It also restores the pool of intracellular reduced glutathione [42] and is an inhibitor of NF-κ B and inflammatory cytokines [43,44]. We used a dose of NAC in the range used in studies in rats and mice [45-47], and found that the dose significantly reduced LHP in the 50/10 OIR model compared to PBS control. However, neither early nor late NAC-treatment groups had significantly reduced avascularity or reduced clock hours of IVNV compared to PBS-injected OIR groups. The clinical efficacy of NAC has not been universally established in vivo. In a clinical trial testing a low dose of NAC (16-32 mg/kg/day) given to premature infants under 1000 g birthweight for the first 6 days after birth, there was no significant difference in survival, body weight gain, respiratory disease, or ROP [48]. Although the authors suggested that too short an intervention was one possible reason for failure, we also did not find an effect using two different treatment regimens and with a higher dose of NAC that we had evidence inhibited endogenous LHP production in the retina. In a review of the efficacy of NAC as a treatment of chronic obstructive pulmonary disease, the authors discussed the discordance in the effects of NAC in in vitro and in vivo studies [49]. Although the reasons remain mostly unknown, NAC can have different effects, particularly as an anti-inflammatory agent, based on dose and cell type and may explain why beneficial effects in vitro are not always translated into the in vivo condition [49]. We found that NAC reduced LHP generation most likely through its effects as an anti-oxidant. However, NAD(P)H oxidase can cause oxidative stress induced apoptosis or inflammation through pathways that are upstream from the generation of LHP, and this may account for our findings. Armstrong et al reported that exogenously injected LHP into rabbit vitreous caused a cascade of inflammatory and angiogenic factors within the retina in a time course associated with the development of IVNV [26,50]. However, there may be differences in tissue responses to exogenously delivered LHP into vitreous from that generated endogenously within the retina. In addition, a recent study of gene expression profiles showed that colon cells exposed to endogenously generated lipid peroxides could mount a DNA repair response whereas those exposed to exogenously administered lipid peroxides could not [51].

NAD(P)H oxidase provides a major source of superoxide radicals during hyperoxia [52] and hypoxia [39] and can trigger pathways leading to apoptosis, VEGF expression, endothelial cell migration, or inflammation [30,39]. Apocynin specifically blocks the activity of NAD(P)H oxidase by interfering with the assembly of the cytosolic NAD(P)H oxidase components (p40phox, p47phox, p67phox) with the membranous components gp91^phox^ and p22phox [39,53,54]. It is also used as an anti-inflammatory drug [55,56]. We first investigated whether there was a peak expression of gp91^phox^ protein in the 50/10 rat OIR model. We found a low expression of gp91^phox^ that did not change significantly from p13 to p18 in the OIR model. However, by immunohistochemistry, we found cells stained for gp91^phox^ antibody within blood vessels in locations of endothelial cells and some leukocytes. Pericytes in vitro have been suggested to express certain homologs of NAD(P)H oxidase [30]. However, the location of gp91^phox^ expression in cryosections of eyes from the OIR model was mainly within CD31 stained endothelial cells and not within NG2 stained pericytes. Some cells may be leukocytes based on co-staining with CD68. Other investigators found higher gp91^phox^ expression in the mouse OIR model than what we found in the rat 50/10 OIR model [32]. This difference may represent differences between species or the model. The mouse OIR model exposes mice to higher and constant oxygen stresses (75% O_2_ for 5 days) [17], whereas the 50/10 OIR model exposes pups to less extreme hyperoxia and not at a constant level, more consistent with blood oxygen levels experienced by preterm infants today. In addition, even a relevant change in protein expression within a small population of cells might have been missed by western blot analysis of a whole retina.

Apocynin injected groups had significantly reduced peripheral retinal avascular areas compared to PBS groups. However, IVNV scores did not differ between apocynin and PBS-injected groups. We did not find a difference in VEGF expression determined by ELISA of tissue samples in apocynin-treated and PBS controls, nor a change in capillary density of newly formed vessels within the vascularized retinas. These findings did not support altered VEGF production as the mechanism for the apocynin effect although a significant change in VEGF expression in a limited number of cells could be missed in analysis of an entire retina by western blot. In addition, the time of assay may have missed a change in VEGF expression. In contrast, by western blot, cleaved caspase-3 was reduced significantly in apocynin-injected groups compared to PBS-injected groups at p14, showing a reduction in apoptosis in association with reduced avascular retina. NAD(P)H oxidase can cause apoptosis indirectly through the release of reactive oxygen species or more directly by activating proapoptotic signaling proteins [30]. The reduction in avascular retina we found from apocynin may be through more direct effects on apoptosis signaling or through apocynin's effects on inflammatory pathways given the variable in vivo responses of NAC as an anti-inflammatory agent [49].

We also stained cryosections for CD68 since NAD(P)H oxidase can trigger inflammatory pathways and found that CD68 labeled cells were co-labeled with gp91^phox^ and rarely with cleaved caspase-3. CD68 labeled leukocytes aligned with lectin-stained vessels in vascularized retina. We did not find CD68 positive cells in the avascular retina where there were no endothelial cells or blood vessels, supporting other studies that show macrophages in newly vascularized retina to be involved in vascularization and remodeling of vessels [57-59]. We found that some lectin stained blood vessels and NG-2 positive perivascular cells co-labeled with cleaved caspase-3 providing support that endothelial cells [60] and pericytes were apoptotic. We also found a number of cleaved caspase-3+ cell nuclei in the inner nuclear and ganglion cell layers, some of which co-labeled with GFAP, likely representing glia. The immunohistochemical studies provided important qualitative co-labeling data that complements the western blot data.

We propose that oxygen fluctuations, such as what premature infants experience [23], cause cells within newly formed blood vessels, mostly endothelial cells and some intravascular leukocytes to express NAD(P)H oxidase, which then leads to increased reactive oxygen species [39]. Through NAD(P)H oxidase activation and either indirectly through ROS generation, inflammatory cytokine production, or more directly by activating proapoptotic signaling proteins [30], endothelial cell and pericyte apoptosis is increased contributing to the avascular retina. In addition, apoptosis of some inner nuclear layer Mueller cells or astrocytes may reduce the production of VEGF, which is necessary in the survival of nascent blood vessels [58]. Investigators have reported that glia, including astrocytes [61] in development and Mueller cells in other OIR models, express VEGF. Although we did not find a difference in VEGF expression in retinas from apocynin- or PBS-treated pups, again a significant difference in a few cells may have been missed in whole retina analyses.

This study provides support that induced NAD(P)H oxidase appears detrimental to newly developed blood vessels within the retina and involves a pathway leading to apoptosis that is associated with avascularity. In the OIR model, endothelial cells express gp91^phox^ and these cells and perivascular NG2 staining pericytes also become apoptotic. Since NAC inhibits most oxidative compounds but did not have an effect on IVNV or avascular retina in the model, our data suggest that the cause of apoptosis may involve a connected but separate pathway from oxidative stress. The anti-inflammatory properties of NAC in vivo have not been consistent. Perhaps, NAD(P)H oxidase is acting through its inflammatory effects or more direct effects on proapoptotic signaling. Reduction of the retinal avascularity may lead to better outcomes by increasing the areas of retinal development and reducing the stimulus for IVNV in ROP.
